# Unveiling the Link Between Celiac Disease and Premature Graying of Hair: A Case Report and Discussion on the Potential Implications for Diagnosis and Management

**DOI:** 10.7759/cureus.65612

**Published:** 2024-07-28

**Authors:** Omar Alomari, Arzu Karaçelik, Elif Rana Tatar, Betul Ulya Durmus, Ozcan Keskin

**Affiliations:** 1 Medicine and Surgery, Hamidiye International Faculty of Medicine, University of Health Sciences, Istanbul, TUR; 2 Internal Medicine, Kartal State Hospital, Istanbul, TUR; 3 Internal Medicine, Kartal Dr. Lutfi Kirdar City Hospital, University of Health Sciences, Istanbul, TUR; 4 Pathology, Kartal Dr. Lutfi Kirdar City Hospital, University of Health Sciences, Istanbul, TUR

**Keywords:** malnutrition, autoimmune disorder, hair color changes, premature graying, celiac disease

## Abstract

Celiac disease (CD) is a systemic autoimmune disorder triggered by gluten ingestion in genetically predisposed individuals and characterized by diverse clinical presentations. Despite its prevalence, CD often remains undiagnosed due to its heterogeneous symptoms and inadequate awareness. Here, we present a case of a 42-year-old male with gastritis who presented with epigastric discomfort and pancytopenia. Initial investigations revealed a hemoglobin level of 3.7 g/dL, a mean corpuscular volume (MCV) of 84 fL, a white blood cell count (WBC) of 2420 cells/μL, a neutrophil count (NEU) of 1400 cells/μL, and a platelet count (PLT) of 140,000 cells/μL. A diagnostic workup revealed evidence of CD, and after that, the diagnosis was confirmed by gastro-colonoscopy. The patient's subsequent adherence to a gluten-free diet resulted in significant clinical improvement. Notably, during follow-up appointments, a notable change in the patient's hair color was observed, prompting further inquiry. The patient reported experiencing premature graying of hair during his late thirties, which remained unchanged until the diagnosis of CD and the initiation of a gluten-free diet. This unique manifestation highlights the potential association between CD and premature graying of the hair, warranting further investigation. While the precise mechanism remains unclear, it is plausible that CD-induced malabsorption and nutritional deficiencies may contribute to such changes. Therefore, we advocate for increased awareness and international collaboration to enhance understanding of this phenomenon and its implications for CD management. This case underscores the importance of early diagnosis and management of CD, as well as the potential for dietary interventions to alleviate associated symptoms and complications.

## Introduction

Celiac disease (CD) is an autoimmune condition distinguished by a distinct serological and histological profile, incited by the ingestion of gluten in genetically susceptible individuals [1]. Celiac disease is additionally recognized as a systemic disorder, manifesting with a variable amalgamation of gluten-related signs, symptoms, and disease-specific antibodies, alongside enteropathy [1]. Celiac disease stands as one of the most prevalent autoimmune disorders, with a reported prevalence ranging from 0.5% to 1% of the general population [2]. Studies indicate that a significant portion of CD cases go undiagnosed without serological screening, largely due to the heterogeneous nature of symptoms and/or inadequate disease awareness [3]. Moreover, the prevalence of CD is on the rise in Western countries [3]. One of the key challenges in diagnosing CD lies in its evolving clinical presentation, which has shifted over time from predominantly childhood symptoms of malabsorption to milder multi-organ manifestations affecting both pediatric and adult patients. This evolution underscores the systemic nature of CD and highlights the need for heightened vigilance in recognizing its diverse manifestations [4]. Celiac disease causes malabsorption, which results in malnutrition, vitamin and mineral deficiency, or insufficiency. This situation affects the whole body, especially the cells with rapid turnover, such as hair, and mostly ends up with rapid hair loss [5]. Additionally, the literature has documented two cases where premature graying of the hair has been linked to CD [6]. Currently, there is no established link between premature graying of the hair and the subsequent diagnosis of adult CD. Herein we report a case in which the patient's hair color changed completely during the late third decade and remained unchanged until the diagnosis of adult CD and adherence to a gluten-free diet. This case report aims to shed light on a unique manifestation of CD and its response to treatment. By documenting this case, we seek to contribute to the understanding of CD's diverse clinical presentations and underscore the potential for dietary intervention to alleviate associated symptoms.

## Case presentation

A 42-year-old male patient, previously diagnosed with gastritis, presented to the emergency department on June 6, 2023, with complaints of an epigastric burning sensation that had persisted for four days. Initial investigations revealed a hemoglobin level of 3.7 g/dL, a mean corpuscular volume (MCV) of 84 fL, a white blood cell count (WBC) of 2420 cells/μL, a neutrophil count (NEU) of 1400 cells/μL, and a platelet count (PLT) of 140,000 cells/μL (Table 1).

**Table 1:** The patient’s initial hematological investigation results with normal reference ranges

Test	Result	Normal reference range
Hemoglobin (Hb)	3.7 g/dL	13.5-17.5 g/dL (men) / 12-15.5 g/dL (women)
Mean corpuscular volume (MCV)	84 fL	80-100 fL
White blood cell count (WBC)	2420 cells/μL	4,000-11,000 cells/μL
Neutrophil count (NEU)	1400 cells/μL	1,500-8,000 cells/μL
Platelet count (PLT)	140,000 cells/μL	150,000-450,000 cells/μL

Notably, there was no history of smoking, gastrointestinal bleeding, active bleeding, or usage of antiplatelet, anticoagulant, or non-steroidal anti-inflammatory drug (NSAID) medications. Additionally, a digital rectal examination showed no abnormalities. Due to the presence of pancytopenia, the patient was admitted for further evaluation. A comprehensive medical examination was conducted, followed by diagnostic imaging, including three-phase contrast-enhanced CT scans. The abdominal CT scan revealed that the liver's size and contour were normal, although there was diffuse, slightly heterogeneous contrast in the liver parenchyma, possibly indicative of geographic fatty infiltration. The pelvic CT scan showed a small amount of free fluid in the pelvis. The CT scan of the thorax identified several nodules in both lungs, with the largest nodule measuring 6 mm in the right lower lobe, warranting follow-up. The neck CT scan did not detect any pathology.

**Figure 1:**
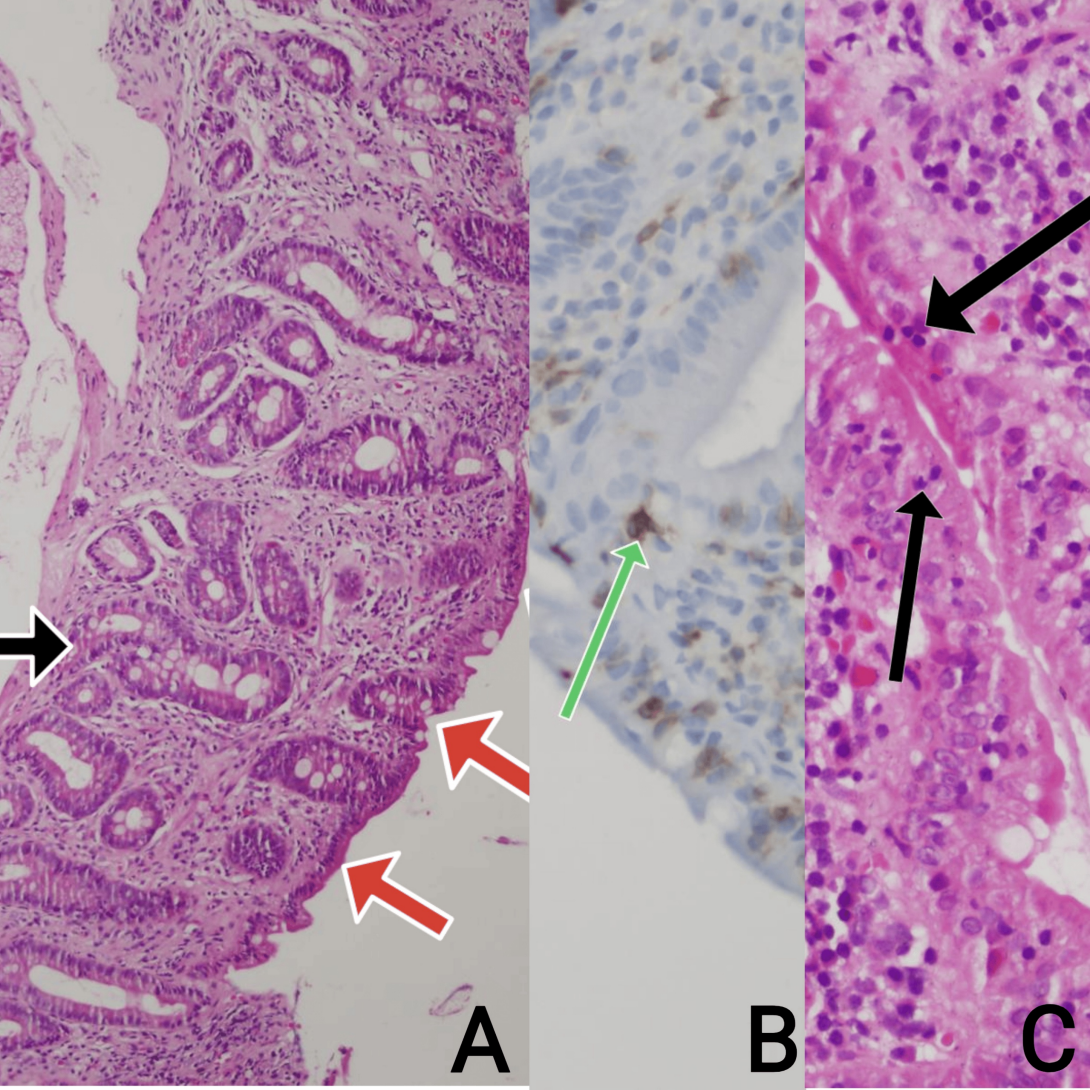
Histological examination of the obtained biopsy A: (duodenum-H&Ex10), marked villus atrophy (red arrows) and crypt hyperplasia (black arrow); B: (duodenum-CD3x40), lymphocyte (green arrows) staining showing infiltration; C: (duodenum-H&Ex40), intraepithelial lymphocytosis (black arrows indicate lymphocytes).

A gastro-colonoscopy was scheduled for the following week, along with laboratory tests for celiac markers. In light of low vitamin B12 and folate levels, the patient was commenced on supplementation, and plans for a blood transfusion were made. Continuous vitamin supplementation and dietary adjustments were implemented, and arrangements were made for gastroscopy and colonoscopy procedures. Further assessments uncovered indications of gluten enteropathy during gastroscopy and the conclusive diagnosis of CD (Marsh classification: 3b) as established following the histological examination of the biopsy sample obtained (Figure 1).

**Figure 2:**
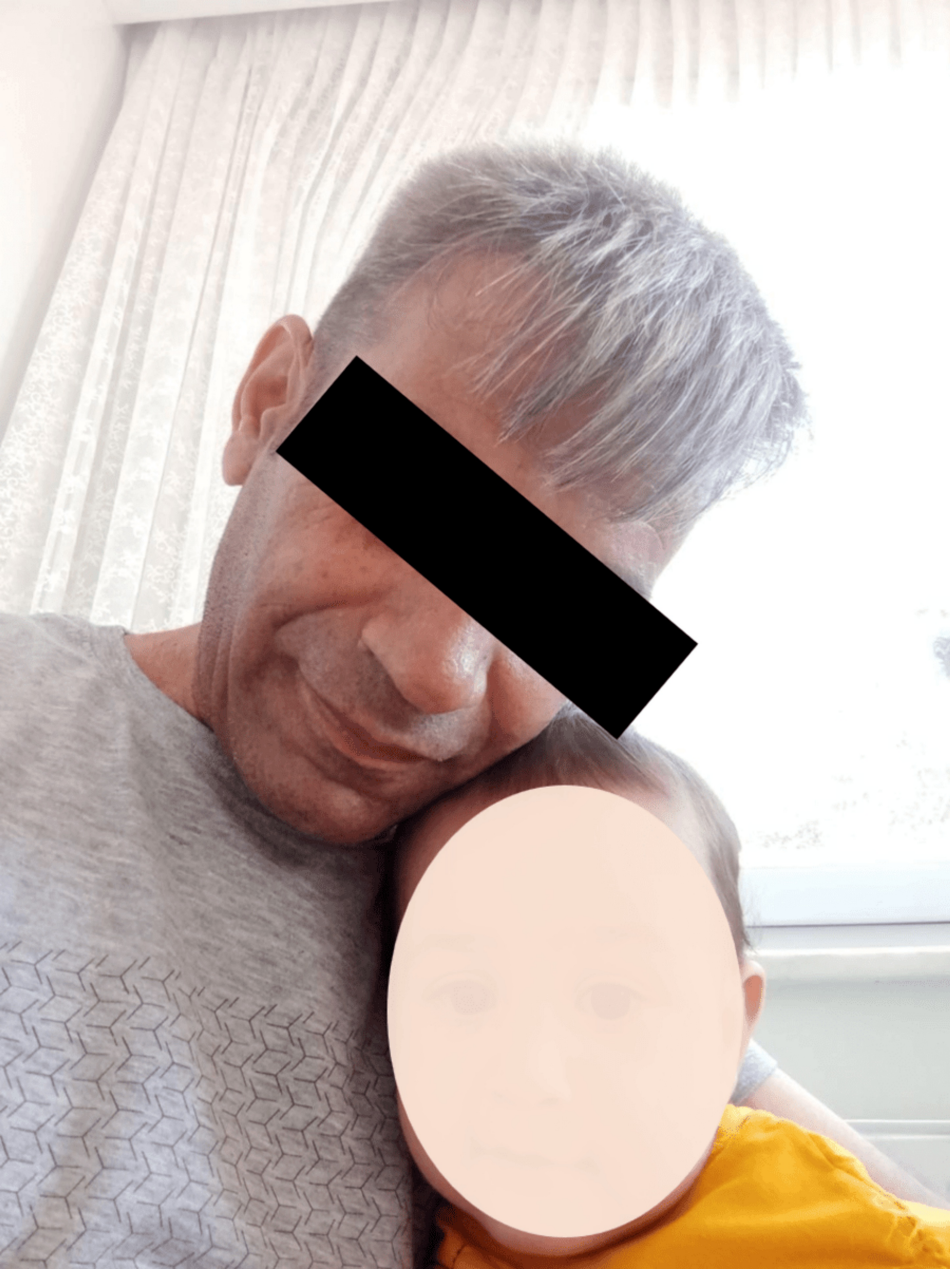
Image showing the hair color of the patient prior to adopting a gluten-free diet The patient was suffering from severe malnutrition and anemia, which significantly affected his appearance.

**Figure 3:**
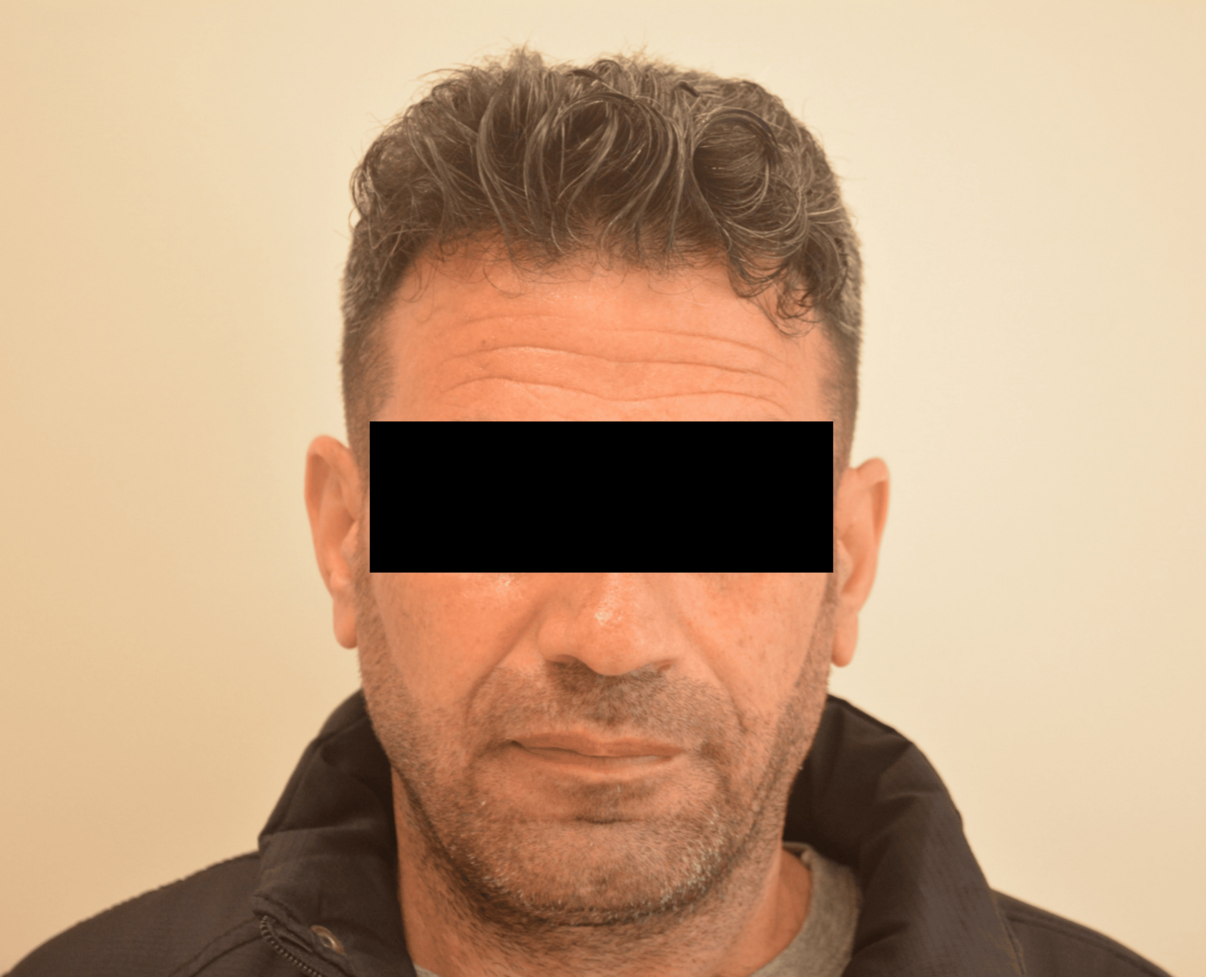
Image of the change in our patient's hair color following adherence to a gluten-free diet Following the treatment, the patient's condition improved dramatically, with noticeable changes in face color and a weight gain of 7 kg by the last visit.

Follow-up appointments and imaging were scheduled according to clinical recommendations. The patient was discharged with ongoing dietary management for gluten enteropathy and scheduled follow-up appointments. During the follow-up period, the patient's overall condition has improved, with elevated blood values observed, and all vitals and general conditions have remained stable. Three months later, during the follow-up appointments, it was noted that the patient's hair color had noticeably changed and become darker. Further inquiry revealed that during his late thirties, the patient had experienced premature graying of his hair (Figure 2), which remained unchanged until the diagnosis of adult CD and the initiation of a gluten-free diet (Figure 3). It's noteworthy to mention that there are stark differences between the before and after photos of the patient. Initially, the patient was suffering from severe malnutrition and anemia, which significantly affected his appearance. Following the treatment, the patient's condition improved dramatically, with noticeable changes in face color and a weight gain of 7 kg by the last visit. This transformation highlights the effectiveness of the treatment and the significant improvement in the patient's overall health. 

## Discussion

This report underscores the importance of considering CD as a potential underlying cause in patients presenting with unusual symptoms, such as premature graying of the hair, and highlights the potential benefits of early diagnosis and management of the condition. While the mechanism underlying the hair color changes in this case remains unclear, its association with gluten sensitivity is evident. It is plausible that such premature graying may serve as either an initial indication of adult coeliac disease or as a coincidental predictor of its subsequent development.

Gray hair is commonly perceived as a symbol of aging and diminished vitality. However, changes in hair growth and color due to malnutrition are widely recognized. Although the influence of various micronutrients like biotin, vitamin B12, zinc, copper, selenium, and iron on the development of premature graying has been hypothesized, substantial evidence is lacking due to the absence of well-designed systematic studies. In 2017, a study was undertaken to assess the levels of micronutrients in patients with premature hair graying compared to controls. The results of the study showed a deficiency of vitamin B12 and folic acid in the patients and lower levels of biotin, without any obvious biotin deficiency in the cases [7]. Another study assessed the relationship between serum iron, copper, and zinc concentrations and premature hair graying. According to the results reported, among copper, zinc, and iron, a low serum copper concentration may play a role in the premature graying of hair [8]. Celiac disease is known to cause malabsorption, which results in malnutrition, vitamin and mineral deficiency, or insufficiency. Since the patient has been gluten-free, his intestines are healing, and are able to absorb more of the nutrients needed for healthy hair growth. However, it’s unknown why this premature graying of the hair is not seen in all CD patients. There is still not enough literature (only two cases) on this topic. That’s why we call for an international effort to get a better understanding of the premature graying of hairs among CD patients [6]. 

Smoking has been investigated as an etiological factor in the premature graying of hair. Studies have demonstrated a significant correlation between smoking and the premature graying of hair. This is likely due to the pro-oxidant effects of smoking, which increase reactive oxygen species (ROS) damage to hair follicle melanocytes [9,10]. However, it is noteworthy that our patient is neither a current smoker nor a former smoker, suggesting that other factors, such as genetic predisposition and immune dysregulation, may play a more significant role in his case.

Several immune diseases have been linked to the premature graying of hair. Vitiligo is an autoimmune disorder that causes the loss of melanocytes because the immune system mistakenly attacks the scalp rather than an infection [11]. Alopecia areata, another autoimmune disorder, causes hair to fall out in patches, particularly affecting hair that still has color [11]. This can result in "overnight" graying, where pre-existing gray and white hair becomes more noticeable. Additionally, chronic protein loss due to medical conditions such as kwashiorkor, nephrosis, and celiac disease can also lead to premature graying [11]. These conditions underscore the complex interplay between immune responses and pigmentation changes.

Recently, a study conducted by Harris et al. aimed to investigate the genetic variants that influence the maintenance of melanocyte stem cells (McSCs) and the mechanisms involved in stem cell self-renewal and tissue regeneration using mouse models of hair graying. Their results revealed that heterozygosity for the melanogenesis-associated transcription factor (MITF) exacerbates McSC differentiation and hair graying in mice predisposed to this phenotype [12]. Transcriptome and molecular analyses of Mitfmi-vga9/+ mice identified a novel role for MITF in regulating systemic innate immune gene expression [12]. These findings highlight the critical role of MITF as a suppressor of innate immunity and its consequences on pigmentation, providing insights that may have implications for the autoimmune depigmenting disease, vitiligo. Understanding these genetic and immunological mechanisms is crucial for developing targeted treatments and preventive strategies for premature graying associated with various underlying conditions. Further research in this area is necessary to fully elucidate these complex interactions and translate these findings into clinical practice. Additionally, raising awareness among healthcare providers about the potential link between premature graying and underlying systemic conditions can lead to earlier diagnosis and better management of these disorders, ultimately improving patient outcomes.

## Conclusions

In conclusion, this case report highlights the significance of considering CD as a potential underlying cause in patients presenting with premature graying of the hair and emphasizes the potential benefits of early diagnosis and management of the condition. Early diagnosis and appropriate management of CD can lead to significant improvements in patient outcomes, including the potential reversal of symptoms not typically associated with the disease, such as premature graying of the hair. This case also highlights the broader implications of dietary interventions for managing autoimmune disorders and improving overall health. Increased research and collaboration are crucial to fully understanding and addressing the systemic effects of CD, ultimately enhancing patient care and quality of life.
